# Health-related quality of life and its related factors in patients with systemic lupus erythematosus in southwest Iran: a cross-sectional study

**DOI:** 10.1186/s40359-023-01300-5

**Published:** 2023-09-01

**Authors:** Sakineh Hashemi, Sahar Farahbakhsh, Zahra Aghakhani, Ali MomayezanMarnani, Nazi Hemati, Somayeh Hashemi

**Affiliations:** 1 Internal Medicine, Shiraz University of Medical Sciences, Shiraz, Iran; 2 Occupational Therapy, Zanjan University of Medical Sciences, Zanjan, Iran; 3 Science, York University, Toronto, Canada; 4 Nursing, Isfahan University of Medical Sciences, Isfahan, Iran; 5 Clinical Exercise Physiology, Shiraz University, Shiraz, Iran

**Keywords:** Quality of life, Systemic Lupus Erythematosus, Iranian people

## Abstract

**Background:**

Quality of life (QoL) is an important measure in health assessment. It is impacted by unclear factors in Systemic Lupus Erythematosus (SLE) patients. The study aimed to investigate the factors related to QoL in SLE patients.

**Methods:**

This cross-sectional study was performed on 140 (136 women and four men) Iranian SLE patients of Hafiz Hospital from June 2019 to August 2020. The Lupus Erythematosus Quality of Life Questionnaire (LEQoL) was used to evaluate the quality of life. The patients were evaluated with this questionnaire for four weeks in eight dimensions health, emotional health, body image, pain, planning, intimate relationships, and the burden of others. Related factors of LEQoL were evaluated using multivariable linear regression.

**Results:**

The mean age was 34.09(8.96) years. The total mean QoL Score was 65.5 ± 22.4. The multivariable analysis showed that duration of disease (β:-1.12, 95% CI:-1.44 to -0.79, P:0.001), physical activity(β:-12.99, 95% CI:-19.2 to -6.13, P:0.001), kidney involvement (β:-9.2, 95% CI:-16.61 to -2.79, P:0.03) and skin involvement(β:-8.7, 95% CI:-17.2 to -0.2, P:0.031) were significantly related to the total mean QOL score of SLE patients.

**Conclusion:**

The QoL of Iranian patients with SLE was low. Age and gender can be related to the decrease in the QoL of patients with SLE. Increasing the disease duration, physical activity, kidney involvement, and skin involvement can be related to the decrease in the QOL of Iranian patients with SLE.

## Introduction

Systemic Lupus Erythematosus (SLE) is known as a chronic autoimmune disorder characterized by cycles of disease activity and remission [1]. Young women in the second to fourth decade of their lives are most commonly affected by SLE, but it can occur at any age and in either gender [2]. Lupus often affects multiple organs and typically causes symptoms such as fatigue, fever, and arthritis, which can negatively impact the quality of life (QoL) and daily functioning [2]. In contrast, SLE causes less common but more serious consequences, such as kidney, cardiopulmonary, and brain diseases (psychosis, seizures, stroke, difficulty concentrating), which contribute significantly to the morbidity and mortality of this disease [3]. Approximately all patients experience the disease’s acute and chronic phases [4]. Moreover, disease activity or damage in SLE has been reported together with decreased mental and physical health leading to a poor QoL [5]. The prevalence of SLE in Iran is estimated to be nearly 40 per 100,000, which is higher than in other Asian countries [6].

While the factors related to the QoL are likely to vary across countries and regions due to economic and cultural factors [7–9]. Although these results show that cultural and ecological elements would impact QoL [10], Recent studies have shown that HRQoL is lower in Iranian patients with SLE [11]. H Shakeri et al. [11] showed that patients with SLE had a lower QoL than the healthy population, especially in physical components. In another study, N Darvish et al. [12] showed that patients with SLE had a poorer QoL than the healthy population. They showed that the simultaneous suffering of patients with SLE with other underlying diseases, especially arthritis, and the disease’s longer duration was significantly related to a decrease in the QOL of Iranian patients with SL.

Various factors are related to the low QoL of patients with SLE [13–15]. The common distributors of poor QoL in SLE include fatigue, pain, sleep disorder, and cognitive dysfunction [5, 16]. The Lupus QoL questionnaire emphasizes a number of specific items of QoL, such as sleeping, body image, and physical health, which have yet to be considered in SF-36 [16] uniquely. SLE disease activity, damage, fibromyalgia, and depression are all associated with poor health-related QoL (HRQoL) [7, 17].

Considering the improvement of survival rate, increase in life expectancy, aging of the population, and in line with that, the increase in the prevalence of underlying diseases such as SLE, estimating the QoL of these patients and also knowing the factors related to the QoL of this disease can improve help the QoL of these patients. In addition, the factors associated with the QoL of Iranian patients with SLE are still unclear. Therefore, considering the importance of this issue, the study aimed to investigate the factors related to QoL in Iranian SLE patients. This study’s results can help improve the QoL of SLE patients by knowing the factors associated with SLE and carrying out related interventions.

## Materials and methods

### Study participants and settings

This study was approved by the Ethics Committee of Shiraz University of Medical Sciences with the code of IR.SUMS.REC.1395.138. This cross-sectional study was conducted on 140 Iranian SLE patients admitted to Hafez Hospital in Shiraz Province from June 2019 to August 2020. 173 patients were referred to this center during the study period. Thirty-three participants needed more complete data and were excluded from the study. Informed consent was obtained from all patients.

### Inclusion and exclusion criteria

Inclusion criteria included: SLE patients with SLEDAI less than 4, age > 5 years, follow-up of at least 12 months, and access to patient findings. Patients with a malignant tumor, depression, chronic anxiety, mental disorders, suffering from viral diseases (hepatitis B, C, and HIV), and drug/alcohol addiction were defined as exclusion criteria.

### Data collection

Individual and clinical factors related to the disease, such as kidney involvement, pain, family history of rheumatic diseases, arthritis, disease activity, etc., were assessed through a comprehensive questionnaire. A rheumatologist clinically examined all patients.

### Measurements

An SLE-specific HRQoL questionnaire called Lupus QoL was used in this study. It consisted of two sections. The first was assigned to clinical and personal information, and the second was to Qol questions. The lupus QoL questionnaire was developed and validated by Kathleen McElhone et al. [18]. It has been translated and validated in Iran by Naeime Sadat Hosseini et al. (2013) [16], which to date is the only Lupus-specific questionnaire that is validated in Iran. The Lupus QoL questionnaire contains 34 items in 8 distinctive domains, including physical health (8 items), emotional health (6 items), body image (5 Items), pain (3 Items), planning (3 items), intimate relationships (2 items) and burden to others (3 items) which are evaluated during four weeks [18]. The items are scored based on a 5-point Likert scale. The final scores vary between 0 and 100. 0 and 100 indicate the worst and best quality of life, respectively. The disease activity was measured by the SLEDAI (Disease Activity Index) scores [19]. The kidney involvement was identified through the patients’ records, including protein urea, BUN, and Creatinine. A minimum of 30 min of walking daily was defined as physical activity.

### Sample size calculation

The appropriate sample size for this study, with an estimated effect size of 0.61 for the difference in the average score of QoL in patients with SLE with the control group based on the study of H Shakeri et al. [11] with an alpha error of 5% and a power of 80% Using G Power version 3.1 software by the methodologist, 71 patients were estimated To increase the power of the study, all patients with SLE who referred within the time period were included in the study.

### Statistical analysis

Data were analyzed using SPS version 22 statistical software. Mean, and standard deviation were used to report quantitative variables. Qualitative variables were reported with descriptive statistics (frequency and %). The Shapiro–Wilk test was used to evaluate the normality of the LEQoL score. The mean score for each dimension of LEQoL was estimated. To compare the variables in two groups, under the assumption that the distribution of the variables is normal, the independent t-test was used, and if normality was not established, the Mann-Whitney test was used. The variables with P value < 0.2 in univariate analysis (Table 1) were entered into the study of multivariable analysis with the Beck method. Multivariable linear regression analysis was used to evaluate the related factors of Lupus QoL and control the confounding variables. The standardized β coefficient with a 95% confidence interval was used to estimate the size of the factors. A p < 0.05 was considered statistically significant.

**Table 1:** Characteristics of the Iranian patients with SLE patients

Variable	140 patients with SLE
Age (year) [mean (SD)]	34.09 (8.96)
BMI (kg/m2)[mean (SD)]	25.03 (2.11)
Starting age of disease (year) [mean (SD)]	23.94 (9.50)
Duration of disease (year) [mean (SD)]	10.10 (6.75)
Marital status n (%)	
Married	110(78.57%)
Single	30(21.43%)
Kidney Involvement n (%)	57 (40.70%)
Arthritis and Arthralgia n (%)	100 (70.40%)
Family History of Rheumatism n(%)	66 (46.50%)
Skin Involvement n(%)	30(21.43%)

## Results

A total of 140 SLE patients (34.09(8.96) years), four men and 136 women were assessed in this study. As Table 2 provides descriptive statistics of patient characteristics, the mean starting age of disease was 23.94(9.50) years, and 78.57% were married. The total mean QoL Score was 65.5(22.4). The highest score of SLE patients’ QoL is related to planning, 78.36(25.03), and the lowest score of QoL was related to emotional, 54.70(30.51). (Table 3)

**Table 2:** Total mean score and subscales of LEQoL Score in patients with SLE patients

LEQoL Score	Mean	SD	Range
Physical Health	74.58	21.6	12,100
Pain	70.5	27.1	0,100
Planning	78.36	25.01	0,100
Intimate Relationship	65.99	39.2	0,100
Burden to other	57.32	33.4	0,100
Emotional	54.7	30.5	0,100
Body Image	55.9	36.5	0,100
Fatigue	67.11	26.5	0,100
Total Score	65.5	22.4	10,100

**Table 3:** Comparison of the mean score of QoL based on demographic and clinical characteristics of patients (results of univariate analysis)

Variable	The total mean score of QoL	P value
Age (year) [mean (SD)]		0.35
≤ 30	67.58 (12.9)	
> 30	63.97 (13.5)	
Educational level [mean (SD)]		0.083
Illiterate	68.54 (26.8)	
<Diploma	54.8 (23.16)	
Diploma	65.13 (22.24)	
>Diploma	71.3 (11.8)	
BMI (kg/m2)[mean (SD)]		0.16
≤ 25	64.59 (21.68)	
> 25	60.98 (23.39)	
Duration of disease (year) [mean (SD)]		0.15
≤ 10		
> 10	67.84 (19.47)	
	60.98 (23.39)	
Marital status [mean (SD)]		0.17
Married	61.11 (19.47)	
Single	66.68 (23.12)	
Kidney Involvement (year) [mean (SD)]		0.009
Yes	59.35 (20.91)	
No	69.35 (23.3)	
Arthritis and Arthralgia [mean (SD)]		0.19
Yes	64.4 (23.43)	
No	66.48 (21.6)	
Skin Involvement [mean (SD)]		0.042
Yes	67.64 (21.25)	
No	57.95 (25.51)	

**T-test or Mann-Whitney test was used

### Univariate analysis finding

The total mean score of QOL in patients with kidney disorders was significantly higher than that of patients without (69.35 (23.3) Vs. 59.35 ( 20.91). (P: 0.009) The total mean score of QOL in patients with and without skin involvement was 57.95 (25.51) and 67.64 (21.25), respectively, which was statistically significant. (P: 0.042) There was no significant difference between the patient’s QoL and other characteristics. (Table 1)

### Multivariable analysis finding

The results of multivariable showed that duration of disease (β:-1.12, 95% CI:-1.44 to -0.79, P:0.001), physical activity(β:-12.99, 95% CI:-19.2 to -6.13, P:0.001), kidney involvement (β:-9.2, 95% CI:-16.61 to -2.79, P:0.03) and skin involvement(β:-8.7, 95% CI:-17.2 to -0.2, P:0.031) were significantly related to the total mean QOL score of SLE patients. Examining variables with different dimensions showed that the increase in BMI, longer duration of disease, physical activity, and kidney and skin involvement were significantly related to the decrease in the mean score of the Physical Health domain. The mean score of the planning dimension decreased significantly with increasing duration of disease, physical activity, and kidney and skin involvement. The mean score of the Intimate relationship domain in married people was significantly lower than that of single people (0.003). The mean score of Burden to other sub-scale improved significantly with increasing age. While the average score of this sub-scale decreased with physical activity, increased disease duration, and kidney involvement (p < 0.05), Education Level was related to the average score of body image and emotional dimensions, so the mean score for this dimension was significantly better in people with an education level higher than diploma than in people with less than a diploma.(p < 0.05) (Table 4).

**Table 4:** Factors related to the QoL of SLE patients based on multivariable analysis

Subscale	Β coefficient	Β Standard	95%CI												P value
			Lower	Upper											
Physical Health															
BMI (Kg/m^2^)	-0.56	-0.18	-1.076										-0.051		0.031
Duration of disease(year) [	-1.01	-0.32	-1.52										-0.47		0.001
Physical activity(Yes vs. No)	-12.03	-0.26	-19.57										-4.47		0.002
Kidney Involvement (Yes vs. No)	-6.01	-0.14	-11.3										-0.057		0.046
Skin Involvement (Yes vs. No)	-14.7	-0.28	-23.2										-6.22		0.001
**Pain**															
Duration of disease (Year)	-1.68	-0.42	-2.31										-1.06		0.001
Physical activity(Yes vs. No)	-22.17	-0.39	-31.12										-13.09		0.001
Skin Involvement (Yes vs. No)	-18.4	-0.28	-28.5										-8.25		0.001
**planning**															
Duration of disease(year)	-1.04	-0.28	-1.64							-0.43					0.001
Physical activity(Yes vs. No)	-14.23	-0.27	-23.11							-5.4					0.002
Kidney Involvement (Yes vs. No)	-10.8	-0.22	-19.01							-2.18					0.001
Skin Involvement (Yes vs. No)	-14.2	-0.23	-23.94							-4.35					0.001
**Intimate relationship**															
Married status (Yes vs. No)	-25.53	-0.29	-41.9				-9.16								0.003
**Burden to other**															
Age (Year)	0.74	0.23	0.15						1.33						0.015
Sex(Female Vs. Male)	-32.38	-2.1	-62.9						-1.88						0.038
Duration of disease	-1.46	-0.3	-2.3						-0.61						0.001
Physical activity(Yes vs. No)	-15.83	-0.23	-27.4					-4.12							0.008
Kidney Involvement (Yes vs. No)	-26.1	-0.38	-37.3					-14.88							0.001
**Emotional**															
Education Level (≥ diploma Vs. <diploma)	6.45	0.27	0.15								8.9				0.001
Duration of disease (Year)	-1.35	-0.3	-2.11								-0.6				0.001
Physical activity(Yes vs. No)	-18.7	-0.29	-29.16								,-7.88				0.001
Kidney Involvement (Yes vs. No)	-12.5	-0.2	-22.7								,-2.12				0.018
**Body image**															
Education Level (≥ diploma Vs. <diploma)	9.87	0.28	2.11											10.59	0.013
Duration of disease (Year)	-0.97	-0.18	-1.87									-0.071			0.035
Kidney Involvement (Yes vs. No)	-13.9	-0.19	-26.6									-1.17			0.033
**Fatigue**															
Duration of disease(Year)	-1.4	-0.37	-2.09			-0.78									0.001
BMI (Kg/m2)	-1.08	-0.045	-1.04			-1.09									0.001
Physical activity(Yes vs. No)	-19.33	-0.35	-28.6			-10.2									0.001
Skin Involvement (Yes vs. No)	-11.43	-0.18	-21.19			-0.93									0.033
**Total Score**															
Duration of disease(Year)	-1.12	-0.32	-1.44		-0.79										0.001
Physical activity(Yes vs. No)	-12.99	-0.28	-19.12		-6.13										0.001
Kidney Involvement (Yes vs. No)	-9.20	-0.24	-16.61		-2.79										0.003
Skin Involvement (Yes vs. No)	-8.7	-0.15	-17.2		-0.2										0.031

## Discussion

As one of the chronic autoimmune diseases, SLE has a wide diversity among different ethnic and geographical groups. With the increase in life expectancy, the prevalence of this disease is increasing, which can relate to the QoL of SLE patients [20–22]. Considering the importance of this issue, in this study, we investigated the factors related to the QoL in LS patients.

The results of our study showed the majority of patients were women. Although the ratio of men to women in other studies is also very high and ranges from 8 to 1 to 12, the female-to-male ratio in our study was slightly higher than in other studies, which can be justified due to the demographic characteristics of the studies. Most patients were married, with a median age of 33 years. The mean overall QoL score in patients with SLE was 65.5. F Conti et al. [23], by examining 117 Italian SLE patients (104 women and 13 men) with a mean age of 40.6 years, reported a total mean of LEQoL subscales of 71.21, consistent with our study’s results. In a study in Iran, N Darvish et al. [12], with a cross-sectional survey of health-related QoL in patients with Weber’s systemic lupus erythematosus on 100 patients and 200 controls, showed that the mean age of the patients was 33 years. The mean overall QoL score was approximately 64. The mean score of QoL in all subscales was lower in SLE patients than in the control group, which confirmed the results of our study. McElhone et al. reported a total mean QoL score for three groups of patients consisting of white (71.21 ± 8.47), Asian (70.81 ± 8.64), and Black Caribbean (71.70 ± 9.12) [24], Which was consistent with the results of our study. Our study’s total mean QoL Score for the Iranian population was 68.45 ± 8.89.

Moreover, Yazdany evaluated the subjects from the United Kingdom (71.07 ± 7.64) and the United States (47.30 ± 5.68) [25]. We finally obtained an overall mean of 68.45 ± 8.89. These measures then represent some assumptions suggesting that the LEQoL Questionnaire requires an adjustment for every culture due to national cross-cultural varieties. Secondly, in the case of Lupus patients, QoL domains would be impacted by further factors in different countries. Thirdly, this group of patients was assumed to enjoy a poor QoL compared to healthy people because different service systems and standards existed.

In our study, multivariable analysis showed that longer disease duration, positive renal involvement, and positive skin involvement were related to reducing the mean score of the total QoL of SLE patients. Examining the relationship between the variables in the subscales showed that an increase in BMI, longer disease duration, physical activity, and kidney and skin involvement could be related to a decrease in the mean score of the physical health domain. The increase in disease duration, physical activity, and kidney and skin involvement were significantly associated with a decrease in the mean score of the planning scale. The mean score of intimate relationships was significantly lower in married patients than in single patients. Multivariable analysis showed that longer disease duration, positive renal involvement, and positive skin involvement were linearly related to the reduction of the average score of the total QoL of SLE patients. Examining the relationship between the variables in the subscales showed that an increase in BMI, longer duration of the disease, positive physical activity, and kidney and skin involvement could be related to a decrease in the average score of the physical health domain. The increase in the duration of illness and physical activity and kidney and skin involvement was significantly associated with a decrease in the mean score of the planning scale. In two subscales, Emotional and Body Image, the mean score of QoL was better in patients with an education level higher than a diploma compared to those with less than a diploma, and the results of our study were consistent with the results of studies conducted in this field [12, 26–29].

In a regular review study by Y Shi et al. [29] in 2021, by evaluating the factors related to the QoL of SLE patients, they showed that SLE patients had poor to moderate QoL. In addition, they showed that damage and involvement in other organs were significantly associated with a decrease in the overall QoL and all patients’ subscale scores, which confirmed our study’s results. In our study, renal and skin involvement were associated with a decrease in the overall mean score of QoL and the mean score of all subscales. M Jeong et al. [30], in 2020, showed that high BMI, lower education level, comorbidities, and involvement in other organs, such as kidney involvement, were significantly associated with decreased QoL in patients with SLE, which was consistent with the results. Our study was consistent. Similar to our study, they used multivariate linear regression analysis to finalize the results. NT Ratanasiripong et al., [31] in Thailand, by examining the factors related to the QoL of SLE patients, showed that skin involvement and the number of symptoms, stress, depression, and anxiety were related to the reduction of the QoL of SLE patients. In our study, skin involvement was associated with decreased QoL of SLE patients. In our study, the effect of anxiety, depression, and stress on QoL was not investigated. C Elera-Fitzcarrald et al., [32] showed that poverty, lower educational level, behavioral issues, some clinical manifestations or skin involvement, and comorbidities were associated with low QoL in SLE patients. In our study, the level of education, skin involvement, and comorbidities was related to low QoL. In our study, the poverty and income level were not investigated, which can be a weak point for our study. In line with the results of our study, S Emamikia et al. [33] showed that the duration of the disease was related to the low QoL of SLE patients.

Also, our study showed that BMI was significantly associated with a decrease in HRQoL in aspects of physical health and fatigue in Iranian SLE patients. In line with the results of our study, A Gomez et al.,(34) in 2021 showed that BMI higher than normal in patients with SLE was associated with a clinically significant decrease in HRQoL in physical aspects and fatigue.

Our study had strengths and weaknesses that should be mentioned. Due to the study design, we could not compare the QoL of SLE patients with the healthy group. Also, in this study, we did not examine a number of important variables, such as income level, anxiety, stress, and depression, that can affect the estimation of the results. In addition, the measurement of many variables, such as physical activity, was based on self-reporting. The design of prospective case-control studies can help estimate the effects more precisely. The most important strength of this study was the investigation of factors predicting QoL in different sub-dimensions in a suitable sample size of Iranian patients with SLE.

## Conclusions

Our study showed that disease activity, kidney involvement, skin involvement, Physical activity, and disease duration are the five principal factors in low QoL. Different numbers of affected participants in the same research would lead to various mean QoL.

## Data Availability

The datasets used and analyzed during the current study are available from the corresponding author on reasonable request.

## List of abbreviations

QoL: Quality of life
SLE: Systemic Lupus Erythematosus
LEQoL: Lupus Quality of Life
HRQoL: Health-related Quality of Life
